# Supplementary material for: The genome sequence of the poplar hawk-moth, Laothoe populi  (Linnaeus, 1758)
Source: Wellcome Open Res. 2025 Mar 18;6:237. Originally published 2021 Sep 16. [Version 2] doi: 10.12688/wellcomeopenres.17191.2 (PMC11937781; doi:10.12688/wellcomeopenres.17191.2)
Supplement: Peer review history

# Reviewer report: Reviewer response for version 2

**DOI:** 10.21956/wellcomeopenres.26312.r120893

**Published:** 2025-04-03

**Authors:** Sivasankaran Kuppusamy

**Affiliations:** 1 Loyola College, Chennai, Tamil Nadu, India

Authors have done the chromosome-level genome assembly of *Laothoe populi *(Linnaeus, 1758). They have assessed the genome of 576 megabases in size.

**Minor comments on the manuscript**

- In the introduction first sentence can be written as “ *Laothoe populi* (Popular hawk-moth) (Linnaeus, 1778) is one of the largest native lepidopteran species in the UK. Its larval colouration varies……
- Last sentence of the introduction can be written as “Here we present a chromosomal-level genome assembly of *L. populi*, based on a female specimen from Wytham Woods, Oxfordshire, UK”.
- The authors have mentioned the estimated genome size 572,54 Mb. I think the estimated genome size may be changed to 572.54 Mb.
- Authors haven’t detected the number of protein-coding genes, number of non-coding genes and number of gene transcripts in the annotation.
- Above all, I confirm that the manuscript meets the necessary scientific standard and is suitable for indexing"

Are sufficient details of methods and materials provided to allow replication by others?

Yes

Is the rationale for creating the dataset(s) clearly described?

Yes

Are the datasets clearly presented in a useable and accessible format?

Yes

Are the protocols appropriate and is the work technically sound?

Yes

Reviewer Expertise:

Phylogeny of Lepidopteran moths

I confirm that I have read this submission and believe that I have an appropriate level of expertise to confirm that it is of an acceptable scientific standard.

# Reviewer report: Reviewer response for version 2

**DOI:** 10.21956/wellcomeopenres.26312.r120895

**Published:** 2025-03-25

**Authors:** Bin Zhang

**Affiliations:** 1 China-Australia Joint Institute of Agricultural and Environmental Health, Qingdao Agricultural University, Shenzhen, China

In the methods section, when describing DNA fragment size analysis, it might be helpful to clarify whether the FemtoPulse analysis was performed on DNA extracted from the homogenate or on the purified DNA.

 Although the revised manuscript includes responses to previous concerns, a brief summary in the discussion regarding the potential biological implications (e.g., the observed interactions between the W chromosome and autosomes) would add context to the technical achievements.

 This revised version represents a significant improvement over the initial submission. The additional details on k-mer profiling, sex chromosome assignment, and the polishing step are welcome. With minor further clarifications—especially regarding parameter details and figure interpretations—the manuscript will be a valuable contribution to the Darwin Tree of Life project and the broader field of Lepidoptera genomics.

 I recommend the manuscript for indexing pending minor revisions addressing the above points.

Are sufficient details of methods and materials provided to allow replication by others?

Yes

Is the rationale for creating the dataset(s) clearly described?

Yes

Are the datasets clearly presented in a useable and accessible format?

Yes

Are the protocols appropriate and is the work technically sound?

Yes

Reviewer Expertise:

Bioinformatics

I confirm that I have read this submission and believe that I have an appropriate level of expertise to confirm that it is of an acceptable scientific standard.

# Reviewer report: Reviewer response for version 1

**DOI:** 10.21956/wellcomeopenres.18994.r46904

**Published:** 2021-11-22

**Authors:** Petr Nguyen

**Affiliations:** 1 University of South Bohemia, Ceske Budejovice, Ceske Budejovice, Czech Republic

The authors report a chromosome-level assembly of the female poplar hawk-moth, *Laothoe populi*, sequenced by PacBio HiFi and 10x Genomics technologies. There is no clear reason for sequencing the genome of *L. populi* as the species has not been of any particular interest to researchers.

 The *L. populi* assembly is 576 Mb long. The *L. populi* genome size is not known but the assembly length falls well within the genome size range of other representatives of the family Sphingidae ( www.genomesize.com). Some comparison with a k-mer based estimate would be nice. More than 99.9% of the sequence was assigned to 29 chromosome-level scaffolds, which correspond to 27 autosomes and sex chromosomes W and Z. This is in agreement with a haploid chromosome number n=28 reported for the species (Robinson 1971, Lepidoptera genetics). The sex chromosomes were probably identified based on their coverage (Figure 2) and Hi-C contact map (Figure 4), but this is not clear from the text and could be explained in more detail in figure legends. Based on Figure 4 it seems that the W chromosome interacts with some of autosomes. I wonder whether it could point to some misassemblies or there is some interesting biology involved. The autosomes are numbered by their size which will be problematic and confusing in future comparative studies. Numbering based on gene synteny with species with ancestral chromosome number n=31 would be preferable.

 The methods section describes the pipeline but not the parameters used. For better reproducibility, parameters used for individual processing and assembly steps should be specified. I believe a tool used for polishing is missing.

Minor comments:

 In methods, “The specimens were snap-frozen” should read “The specimen was snap-frozen” as only one female was collected.

 I believe the sentence “Fragment size analysis of 0.01-0.5 ng of DNA was then performed using an Agilent FemtoPulse” should follow DNA extraction. Or was the fragment size analysis performed with crude homogenate?

Are sufficient details of methods and materials provided to allow replication by others?

Partly

Is the rationale for creating the dataset(s) clearly described?

Yes

Are the datasets clearly presented in a useable and accessible format?

Yes

Are the protocols appropriate and is the work technically sound?

Yes

Reviewer Expertise:

Genetics of Lepidoptera

I confirm that I have read this submission and believe that I have an appropriate level of expertise to confirm that it is of an acceptable scientific standard, however I have significant reservations, as outlined above.

# Response

**Published:** 2025-02-19

**Authors:** Tree of Life Team Sanger

**Affiliations:** Wellcome Trust Sanger Institute, UK

Thank you for reviewing this data note. We have addressed your comments in version 2 as follows:

- The *k*-mer profiles of the raw reads were generated in GenomeScope prior to assembly. In version 2 of this data note we have included details of this method and the resulting genome size estimate.
- Information about the assignment of sex chromosomes has been added. Frequent interactions between the W chromosome and autosomes are known biological phenomena, as documented in the literature, and are not indicative of misassembly.
- In line with standard practice, we have numbered chromosomes by size. This approach maintains consistency, given the chromosomal rearrangements and number changes that can complicate strict synteny-based numbering.
- The polishing tool is mentioned in the text: “ *The assembly was polished with 10X Genomics Illumina data by aligning to the assembly with longranger align and calling variants with freebayes (Garrison & Marth 2012).*”
- We have corrected the wording of the methods - thank you for drawing our attention to these issues.

# Reviewer report: Reviewer response for version 1

**DOI:** 10.21956/wellcomeopenres.18994.r46101

**Published:** 2021-10-18

**Authors:** Roderic Guigo Guigo

**Affiliations:** 1 Centre for Genomic Regulation (CRG), The Barcelona Institute of Science and Technology, Barcelona, Spain

This is a concise, clear, precise note that describes the assembly of a moth species within the Darwin Tree of Life project. I am informed, but not an expert in genome assembly. I believe, however, that the metrics provided show that this is a high quality assembly, well exceeding the VGP/EBP standards.

 As a non-expert in genome assembly, it took me a while to fully understand the information displayed in the Figures - in particular, those generated by the Blob Toolkit. Accessing the interactive version of the figures helped some, but the meaning of the different figure components is not immediately obvious either through the interactive interface. If these plots will become the standard for the DToL genome data notes, it maybe helpful to have a direct link to a description of what is shown in the figures.

 Regarding the static figures, maybe there should be an easy way to show the chromosomal scaffolds, I did not find a way to easily visualize the chromosomes in the snail plot.

Are sufficient details of methods and materials provided to allow replication by others?

Yes

Is the rationale for creating the dataset(s) clearly described?

Yes

Are the datasets clearly presented in a useable and accessible format?

Yes

Are the protocols appropriate and is the work technically sound?

Yes

Reviewer Expertise:

Transcriptomics, Gene finding, comparative genomics

I confirm that I have read this submission and believe that I have an appropriate level of expertise to confirm that it is of an acceptable scientific standard.
